# β-hydroxybutyrate dehydrogenase promotes pancreatic cancer cell proliferation through regulation of the NAD^+^/NADH balance and mitochondrial acetylation

**DOI:** 10.1016/j.jbc.2025.110636

**Published:** 2025-08-28

**Authors:** Xiujuan Wei, Chengkai Zhang, Xiaoguang Zheng, Kexin Pan, Xiaojun Ren, Xiaoting Lou, Zhengquan Yang, Ting Fu, Hezhi Fang, Jianxin Lyu

**Affiliations:** 1 Laboratory Medicine Center, Department of Clinical Laboratory, Zhejiang Provincial People's Hospital, Affiliated People's Hospital, Hangzhou Medical College, Hangzhou, Zhejiang, China; 2 College of Laboratory Medicine and Life Sciences, Wenzhou Medical University, Wenzhou, Zhejiang, China; 3 School of Basic Medical Sciences and Forensic Medicine, Hangzhou Medical College, Hangzhou, Zhejiang, China; 4 School of Laboratory Medicine and Bioengineering, Hangzhou Medical College, Hangzhou, Zhejiang, China; 5 Department of Clinical Laboratory, State Key Laboratory of Molecular Oncology, National Cancer Center/National Clinical Research Center for Cancer/Cancer Hospital, Chinese Academy of Medical Sciences and Peking Union Medical College, Beijing, China; 6 Department of Clinical Laboratory, The Second Affiliated Hospital and Yuying Children's Hospital of Wenzhou Medical University, Wenzhou, Zhejiang, China

**Keywords:** pancreatic cancer, BDH1, ketone body, NAD^+^/NADH, mitochondrial acetylation

## Abstract

Ketone bodies are a key alternative energy source during carbohydrate deficiency. In addition to their metabolic function, they regulate essential cellular processes, including metabolism, signal transduction, and protein post-translational modifications (PTMs). However, the role of ketone body metabolism in tumorigenesis remains poorly understood. Here, we demonstrate that ketone body synthesis metabolism is activated in pancreatic cancer, while exogenous ketone supplementation does not affect PDAC cell proliferation. Moreover, we observe a significant upregulation of β-Hydroxybutyrate dehydrogenase (BDH1), a key enzyme in ketone body metabolism, in human pancreatic ductal adenocarcinoma (PDAC) tissues compared to adjacent normal pancreatic tissues. BDH1 promotes PDAC cell proliferation by maintaining mitochondrial acetylation levels through regulation of the intracellular NAD^+^/NADH ratio. These findings underscore the importance of ketone body metabolism in pancreatic cancer progression and highlight the regulatory role of BDH1 in maintaining cellular NAD^+^/NADH balance and mitochondrial acetylation.

Pancreatic cancer is a highly aggressive gastrointestinal malignancy, often referred to as the “king of cancers” (1). Among its various forms, pancreatic ductal adenocarcinoma (PDAC) is the most common, accounting for over 90% of all pancreatic tumors. PDAC is characterized by its insidious onset, with no distinct symptoms in the early stages, rapid progression, and high malignancy, resulting in a 5-year survival rate of of only 13.7% (2, 3). PDAC tissue consists predominantly of stromal components, which make up more than 90% of the tumor mass (4). This dense stromal matrix creates a hypoxic and nutrient-deprived microenvironment that severely limits the availability of oxygen and nutrients to PDAC cells (5). Research has shown that PDAC cells adapt to this harsh environment through metabolic reprogramming (6). Despite significant advances in understanding these mechanisms over the past decade, important gaps in knowledge remain.

Ketone bodies, including β-hydroxybutyrate (HB), acetoacetate (AcAc), and acetone, are synthesized in the liver from acetyl-CoA produced through β-oxidation of fatty acids when carbohydrate availability is limited (7). These ketone bodies are then transported to other organs and tissues, where they are oxidized and converted back into acetyl-CoA, which enters the TCA cycle to provide energy. Under hypoxic conditions, mitochondrial oxidative phosphorylation (OXPHOS) is inhibited, and the body shifts from using oxygen-dependent substrates to energy sources with higher oxygen efficiency (8). Notably, each mole of ketone body oxidation consumes more oxygen than glucose but produces more ATP. Therefore, the body preferentially utilizes ketone bodies under hypoxic conditions (9).

However, the role of ketone bodies in tumorigenesis is complex, exhibiting both promotive and inhibitory effects (10). The specific outcome depends on various factors, such as tumor type, the tumor microenvironment, and the metabolic state of the cells. For instance, it has been reported that acetoacetate selectively activates the MEK1 pathway in BRAF V600E cells, thereby promoting melanoma progression (11). On the other hand, HB induces the transcriptional regulator HOPX through interaction with the surface receptor HCAR2, leading to altered gene expression and inhibiting the proliferation of colorectal cancer cells (9). Additionally, some studies have shown that a ketogenic diet combined with chemotherapy significantly increases NADH levels in pancreatic cancer cells, synergistically inhibiting tumor growth in mouse models (12). However, other studies suggest that PDAC cells can synthesize HB as an energy source to support their growth and progression (13). Despite these findings, most existing research has focused on the role of ketone bodies as energy substrates or signaling molecules in tumor growth, with limited exploration of the broader impact of ketone body metabolism on tumor initiation and progression. A more comprehensive understanding of these processes is needed.

In this study, we demonstrated that ketone body anabolism is activated in PDAC cells, whereas exogenous supplementation with ketone bodies (HB and AcAc) does not affect their growth. Furthermore, BDH1, a key enzyme in ketone body metabolism, promotes PDAC cell growth by increasing intracellular NAD^+^/NADH levels, decreasing mitochondrial acetylation.

## Results

### Ketogenesis is positively associated with PDAC

**Figure 1:**
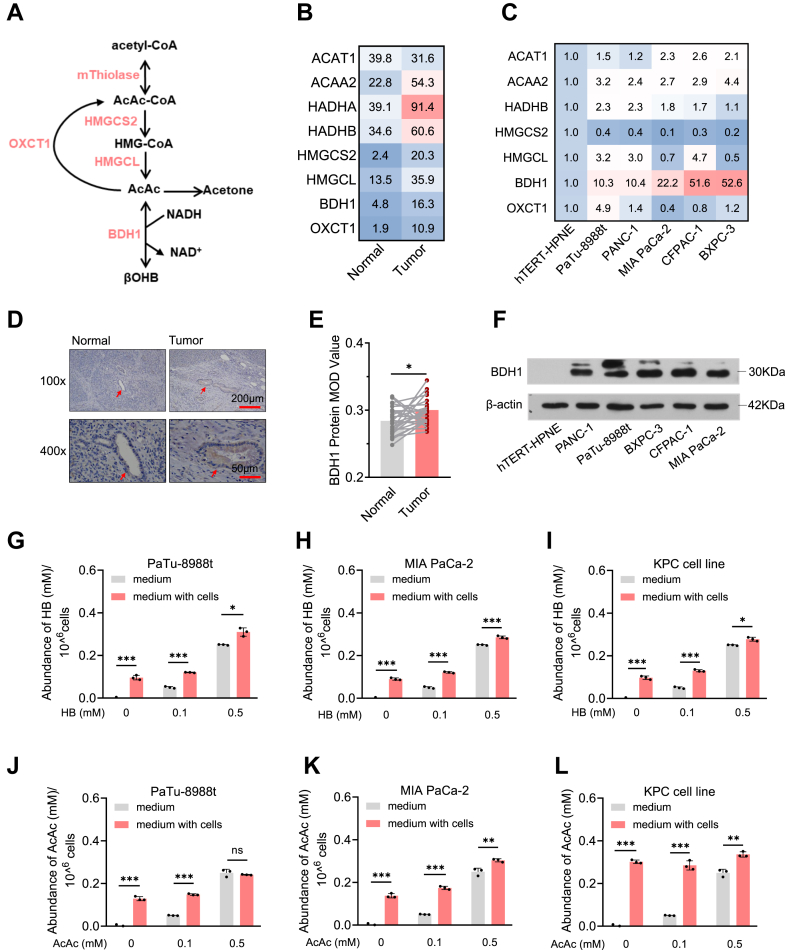
**Ketogenesis is positively associated with PDAC**. *A*, metabolic Pathway of the ketone body, mThiolase: Mitochondrial Thiolase. *B,* mRNA levels of ketone body metabolism-related genes in PDAC (TCGA) and normal pancreatic tissues (GTEx database), normal pancreatic tissues (N = 171), and PDAC tissues (T = 179). *C*, mRNA levels of the ketone body metabolism-related gene in the normal pancreatic ductal epithelial cell line (hTERT-HPNE) and PDAC cell lines (PaTu-8988t, PANC-1, MIA PaCa-2, CFPAC-1 and BXPC-3). *D–E*, immunohistochemical analysis of BDH1 expression was performed on PDAC tissues (n = 27) and matched adjacent normal tissues (n = 27). Representative immunohistochemistry images are shown in panel at 100 × (scale bar = 200 μm) and 400 × (scale bar = 50 μm) magnification (*D*). Quantitative results are presented in *panel* (*E*). *F*, Western blot analysis of BDH1 in the normal pancreatic ductal epithelial cell line (hTERT-HPNE) and 5 PDAC cell lines. *G–I*, HB concentration in the culture medium of PDAC cell lines after 24h treatment with different HB concentrations (0 mM, 0.1 mM, 0.5 mM) under conventional culture conditions, PaTu-8988t (*G*), MIA PaCa-2 (*H*), KPC cell line (*I*). *J–L* AcAc concentration in the culture medium of PDAC cell lines after 24h treatment with different AcAc concentrations (0 mM, 0.1 mM, 0.5 mM) under conventional culture conditions, PaTu-8988t (*J*), MIA PaCa-2 (*K*), KPC cell line (*L*).

Ketone bodies (β-hydroxybutyrate, acetoacetate, and acetone) serve as critical alternative energy substrates under nutritional deficiency, primarily synthesized in the liver and transported through the bloodstream to various tissues for metabolism and utilization. A growing body of evidence suggests that ketone bodies influence the development and progression of various cancers through modulation of cellular energy metabolism, signaling pathways, and post-translational modifications (PTMs) (13). To explore the role of ketone body metabolism (Fig. 1*A*) in pancreatic cancer progression, we analyzed mRNA expression data from pancreatic cancer patients and controls sourced from the Cancer Genome Atlas (TCGA) and Genotype-Tissue Expression (GTEx) databases. Our comparative analysis revealed that ketone metabolism-related genes are generally upregulated in pancreatic cancer tissues (Fig. 1*B*). Further examination of single-cell transcriptomic data from patients with pancreatic cancer showed a significant increase in the expression of most ketone metabolism-related genes in pancreatic cancer ductal cells compared to normal ductal epithelial cells (Fig. S1*A*). This observation was corroborated by single-cell transcriptomic data from the KPC mouse model (Fig. S1*B*). Additionally, qPCR analysis demonstrated that, relative to normal pancreatic epithelial cells (hTERT-HPNE), the expression of all ketone metabolism-related genes, with the exception of *HMGCS2*, was significantly elevated in PDAC cells, with *BDH1* showing the most pronounced increase (Fig. 1*C*). Immunohistochemical staining of paraffin-embedded tissue sections from 27 patients with PDAC was performed to assess BDH1 expression levels. The results showed that BDH1 expression was significantly increased in pancreatic cancer tissue compared to the adjacent normal ductal tissue (Fig. 1, *D* and *E*). Similarly, BDH1 protein levels were notably higher in PDAC cells than in normal pancreatic epithelial cells (Fig. 1*F*).

To verify whether PDAC cells can synthesize ketone bodies, we cultured PDAC cells in a medium prepared with dialyzed, ketone body-free serum and measured the concentrations of HB and AcAc in the culture supernatants. Surprisingly, even in the absence of HB and AcAc in the medium, both ketone bodies were detectable in the supernatants of PaTu-8988t, MIA PaCa-2, and KPC cell lines. When the concentrations of HB and AcAc in the medium were 0.1 and 0.5 mM, respectively, the levels of both ketone bodies increased in the supernatant after PDAC cell culture (Fig. 1, *G*–*L*). Similarly, under physiological concentrations of glucose (5 mM) and glutamine (0.5 mM), we observed similar results (Fig. S1, *C* and *D*). These findings indicate that PDAC cells are capable of synthesizing and releasing ketone bodies.

### Ketone bodies supplementation does not affect PDAC cell proliferation

**Figure 2:**
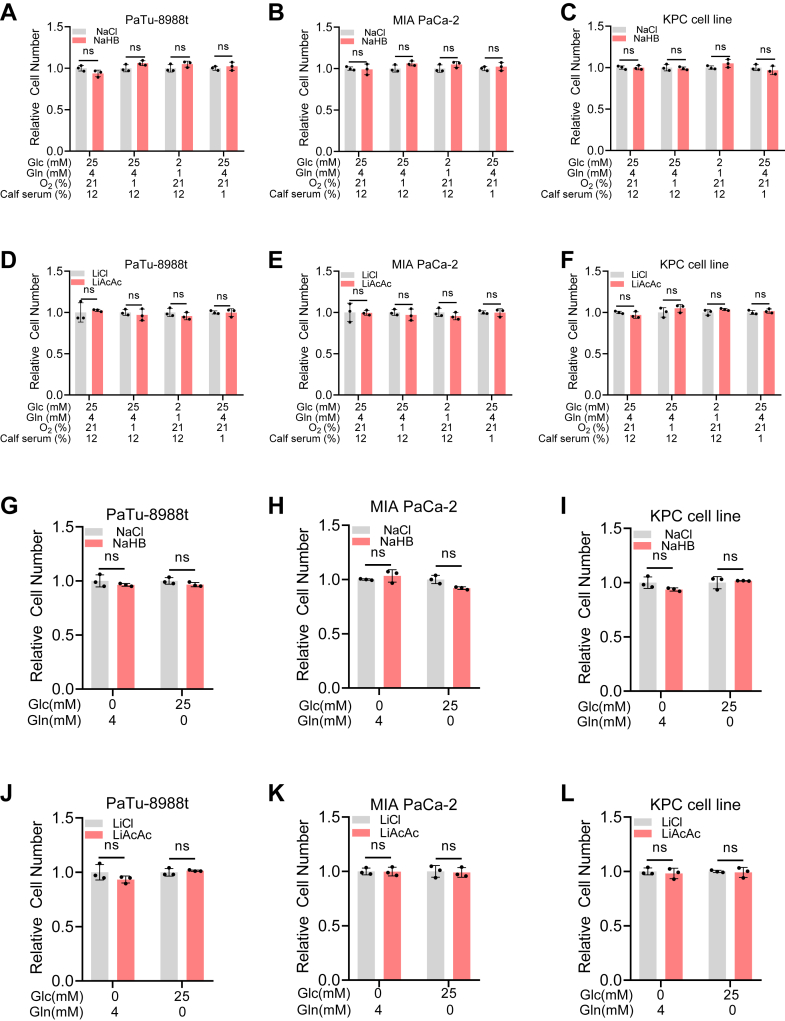
**Ketone bodies supplementation does not affect PDAC cell proliferation**. *A–C*, proliferation of PDAC cells with HB added under high-nutrient, hypoxic, low-glucose/low-glutamine, or low-serum environments, control group: NaCl (2 mM), experimental Group: NaHB (2 mM), Glc: glucose, Gln: glutamine, ns: no significance, PaTu-8988t (*A*), MIA PaCa-2 (*B*), or KPC cell line (*C*). *D–F*, proliferation of PDAC cells with AcAc added under high-nutrient, hypoxic, low-glucose/low-glutamine, or low-serum environments, control group: LiCl (2 mM), experimental Group: LiAcAc (2 mM), Glc: glucose, Gln: glutamine, ns: no significance, PaTu-8988t (*D*), MIA PaCa-2 (*E*), or KPC cell line (*F*). *G*–*I* PDAC cell survival rate in the absence of glucose or glutamine after HB supplementation, control group: NaCl (2 mM), experimental Group: NaHB (2 mM), Glc: glucose, Gln: glutamine, experimental Group: LiAcAc (2 mM), ns: no significance, PaTu-8988t (*G*), MIA PaCa-2 (*H*), or KPC cell line (*I*). *J–L*, PDAC cell survival rate in the absence of glucose or glutamine after AcAc supplementation, control group: LiCl (2 mM), experimental Group: LiAcAc (2 mM), Glc: glucose, Gln: glutamine, experimental Group: LiAcAc (2 mM), ns: no significance, PaTu-8988t (*J*), MIA PaCa-2 (*K*), or KPC cell line (*L*).

Previous studies have presented conflicting views on the role of ketone bodies in pancreatic cancer growth (14). In our study, we tested the ability of PDAC cells to synthesize ketone bodies and found that these cells can indeed produce ketone bodies. This suggests that ketone body anabolism may play a significant role in the initiation and progression of PDAC. First, we confirmed that exogenous ketone bodies could be effectively taken up by cells and increase their intracellular levels by detecting lysine β-hydroxybutyrylation (Kbhb) and intracellular AcAc concentrations (Fig. S2, *A* and *B*). To determine whether HB and AcAc promote PDAC cell growth, we established four distinct culture conditions: high-nutrient, hypoxic, low-glucose/low-glutamine, and low-serum environments. We then assessed the impact of exogenous HB or AcAc on cell proliferation. The results showed that neither HB nor AcAc affected the proliferation of PaTu-8988t, MIA PaCa-2, or KPC cell lines (Fig. 2, *A*–*F*). Additionally, the effects of HB and AcAc on PDAC cell survival were evaluated under glucose- and glutamine-based conditions. Similarly, the addition of HB and AcAc did not significantly alter the survival of these cell lines (Fig. 2, *G*–*L*). In summary, our findings show that HB and AcAc do not promote the proliferation or survival of PDAC cells under various culture conditions. These results suggest that ketone bodies do not serve as effective energy substrates for enhancing the proliferation or survival of PDAC cells.

### BDH1 depletion inhibits PDAC cell proliferation

**Figure 3:**
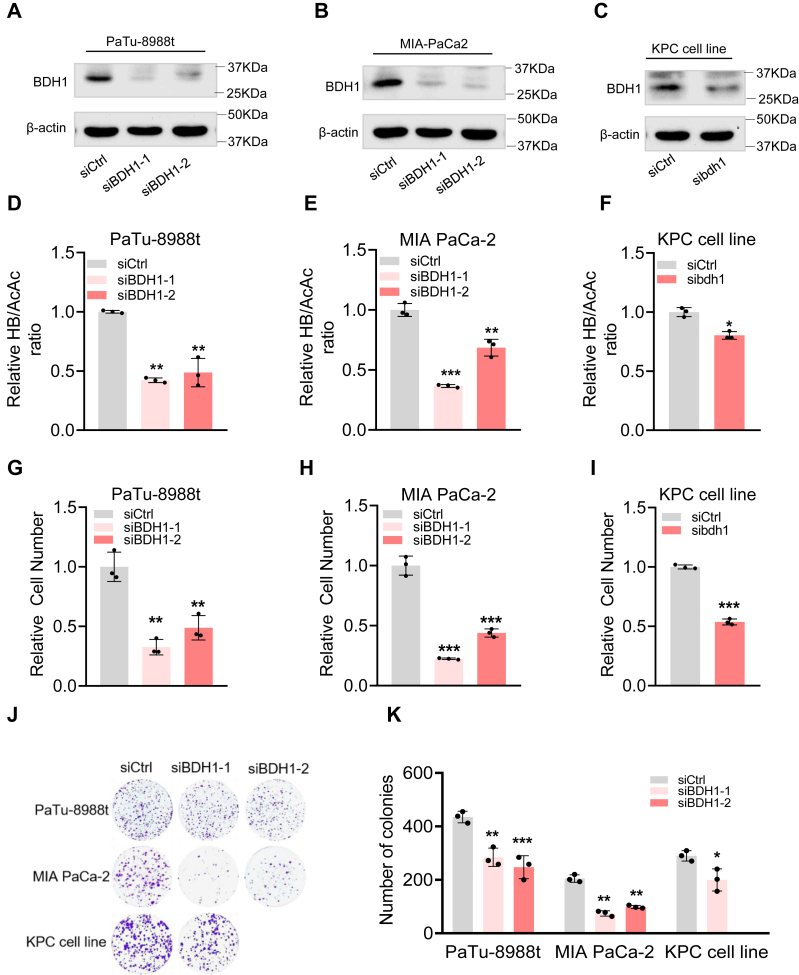
**BDH1 depletion inhibits PDAC cell proliferation**. *A–C*, PDAC cells were transfected with control siRNA or BDH1(bdh1) siRNA, Western blot analysis was performed with the indicated antibodies, PaTu-8988t (*A*), MIA PaCa-2 (*B*), or KPC cell line (*C*). *D–F*, PDAC cells transfected with control siRNA or BDH1 (bdh1) siRNA. HB/AcAc ratio in the culture medium after 24 h of cell culture, relative values were determined by comparing cells transfected with control siRNA to those transfected with BDH1 (bdh1) siRNA (mean ± SD, n = 3), PaTu-8988t (*D*), MIA PaCa-2 (*E*), or KPC cell line (*F*). G-I PDAC cells transfected with control siRNA or BDH1 (bdh1) siRNA were seeded at a density of 1 × 10^4^ cells per well in 12-well plates. After 72 h of culture, relative values were determined by comparing cells transfected with control siRNA to those transfected with BDH1 (bdh1) siRNA at the final time point (mean ± SD, n = 3), PaTu-8988t (*G*), MIA PaCa-2 (*H*), or the KPC cell line (*I*). *J–K*, colony formation assay, PDAC cells transfected with control siRNA or BDH1 (bdh1) siRNA were seeded at a density of 1 × 10^3^ cells/well in 6-well plates and cultured for 10 days (*J*), quantitative statistical results (*K*).

BDH1 is a key enzyme in ketone body metabolism, catalyzing the conversion of HB to AcAc. It represents both the final step in ketone body synthesis and the initial step in ketone body catabolism. To further investigate the role of BDH1 in pancreatic cancer, we used small interfering RNA (siRNA) technology to generate BDH1 knockdown cell lines in PaTu-8988t, MIA PaCa-2, and KPC cell lines (Fig. 3, *A*–*C*). We found that knocking down BDH1 in PaTu-8988t, MIA PaCa-2, and KPC cell lines inhibited the conversion of HB to AcAc, further supporting the ability of PDAC cells to synthesize ketone bodies (Fig. 3, *D*–*F*). Functionally, silencing BDH1 significantly inhibited cell proliferation (Fig. 3, *G*–*I*) and suppressed clonogenic potential (Fig. 3, *J* and *K*) in PDAC cells, highlighting a tumor-promoting role for BDH1. To determine whether the antiproliferative effects of BDH1 knockdown were associated with increased apoptosis, we performed Annexin V/PI staining and flow cytometry analysis. As shown in Figure S3, *A*–*C*, BDH1 knockdown did not significantly induce apoptosis in PaTu-8988t and MIA PaCa-2, and only a modest increase in apoptotic cells was observed in the KPC cell line (Fig. S3, *A*–*C*). These results suggest that BDH1 depletion impairs PDAC cell proliferation primarily through apoptosis-independent mechanisms.

Since HB deficiency is known to decrease β-hydroxybutyrylation modification levels (15), which are associated with cell proliferation, we measured the β-hydroxybutyrylation levels in these PDAC cells. The results confirmed that BDH1 knockdown led to a slight reduction in β-hydroxybutyrylation compared to control cells, a difference that could be rescued by exogenous HB supplementation (Fig. S4, *A*–*C*). To determine whether the decrease in intracellular HB levels contributes to the reduced proliferation of BDH1-deficient cells, we assessed the proliferation of BDH1 knockdown cells following exogenous HB addition. However, HB supplementation did not rescue the proliferative defects observed in BDH1 knockdown cells (Figure S4, *D*–*F*), suggesting that the reduction in intracellular HB is not the cause of the proliferation decrease. Next, to explore whether AcAc accumulation contributes to the reduced proliferation in BDH1-deficient cells, we simulated AcAc accumulation by exogenously adding AcAc. We then assessed the proliferation of both control and BDH1 knockdown cells. The results showed that adding AcAc did not alleviate the proliferation difference between BDH1 knockdown and control cells compared to the addition of lithium chloride. This indicates that AcAc accumulation cannot rescue the reduced proliferation in BDH1 knockdown cells (Fig. S4, *G*–*I*).

### BDH1 is required to maintain NAD^+^/NADH balance *via* regulating ketonegenesis

**Figure 4:**
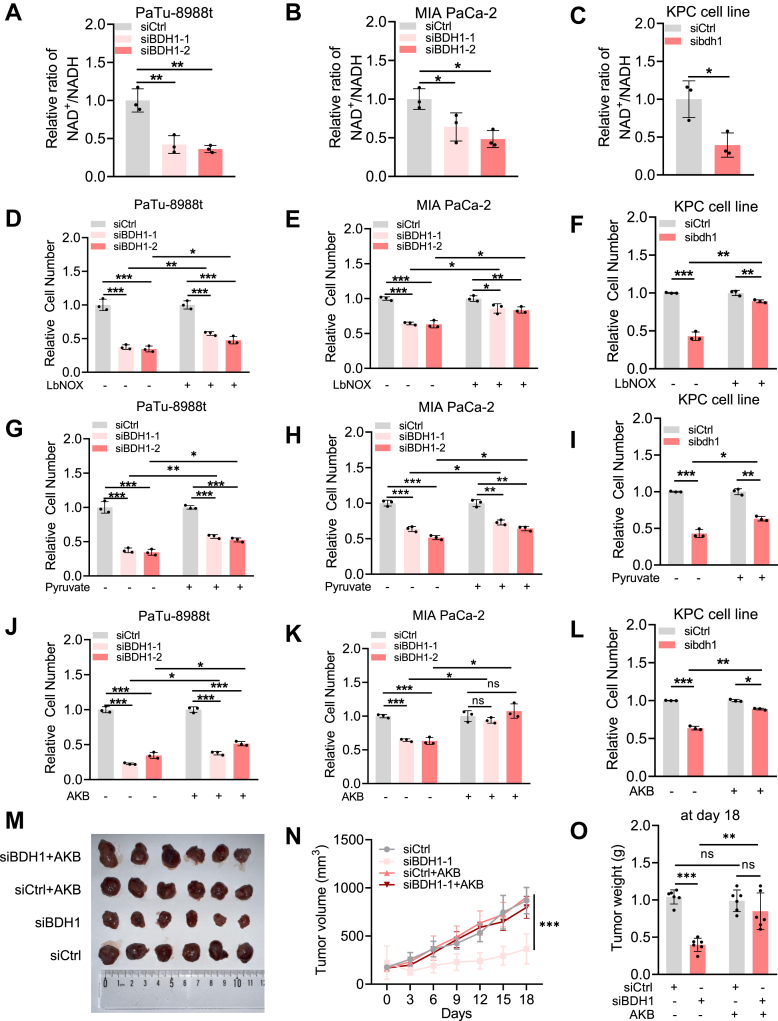
**BDH1 is required to maintain NAD^+^/NADH balance *via* regulating ketonegenesis.***A–C*, PDAC cells transfected with control siRNA or BDH1 (bdh1) siRNA were plated 48 h before determination of cellular NAD^+^/NADH ratio, relative values were determined by comparing cells transfected with control siRNA to those transfected with BDH1 (bdh1) siRNA (mean ± SD, n = 3), PaTu-8988t (*A*), MIA PaCa-2 (*B*), or KPC cell line (*C*). *D–F*, PDAC cells transfected with control siRNA or BDH1 (bdh1) siRNA. Cell proliferation of overexpresing or not LbNOX, relative values were determined by comparing cells transfected with control siRNA to those transfected with BDH1 (bdh1) siRNA (mean ± SD, n = 3), PaTu-8988t (*H*), MIA PaCa-2 (*I*), or KPC cell line (*J*). *G–I*, PDAC cells transfected with control siRNA or BDH1 (bdh1) siRNA.Cell proliferation with and without pyruvate supplementation, relative values were determined by comparing cells transfected with control siRNA to those transfected with BDH1 (bdh1) siRNA (mean ± SD, n = 3), PaTu-8988t (*G*), MIA PaCa-2 (*H*), or KPC cell line (*I*). *J–L*, PDAC cells transfected with control siRNA or BDH1 (bdh1) siRNA. Cell proliferation with and without AKB supplementation, relative values were determined by comparing cells transfected with control siRNA to those transfected with BDH1 (bdh1) siRNA (mean ± SD, n = 3), PaTu-8988t (*J*), MIA PaCa-2 (*K*), or KPC cell line (*L*). *M–O*, PaTu-8988t cells (5 × 10^6^), transfected with either control siRNA or siBDH1, were subcutaneously injected into the axilla of athymic nude mice. One week after injection, once tumors had formed, AKB (50 mg/kg) was administered intraperitoneally every 3 days. Tumor volume (*L*) was measured at each injection time point, and tumor weight (*M*) was recorded after 18 days of growth (mean ± SD, n ≥ 6).

The BDH1-mediated conversion of HB to AcAc is coupled with the interconversion of NAD^+^ and NADH. NAD^+^ serves as a crucial cofactor in numerous cellular metabolic processes (16). Notably, cancer cells exhibit a higher NAD^+^/NADH ratio compared to normal cells, and this ratio plays a pivotal role in cancer initiation and progression (17). To investigate the mechanism by which BDH1 promotes cell proliferation, we measured the NAD^+^/NADH ratio in three PDAC cell lines. Our results revealed a significant reduction in the NAD^+^/NADH ratio in BDH1 knockdown cells compared to control cells (Fig. 4, *A*–*C*). Conversely, BDH1 overexpression in PaTu-8988t cells led to an increased NAD^+^/NADH ratio (Fig. S5, *A* and *B*). LbNOX, a protein from *Lactobacillus brevis*, functions as an oxidative dehydrogenase capable of spontaneously converting NADH to NAD^+^ (Fig. S5*F*) (18). To restore NAD^+^/NADH levels, we overexpressed LbNOX in BDH1 knockdown cells (Fig. S5, *C*–*E*). The NAD^+^/NADH ratio in these cells was restored to levels comparable to control cells, indicating that LbNOX effectively compensates for the reduction in the NAD^+^/NADH ratio caused by BDH1 deficiency (Fig. S5, *G*–*I*). Furthermore, cell proliferation assays showed that LbNOX overexpression partially rescued the proliferation defect in BDH1 knockdown cells across all three PDAC cell lines (Fig. 4, *D*–*F*). Similarly, pyruvate and α-ketobutyrate (AKB) can be reduced to lactate and α-hydroxybutyrate (AHB), respectively, by lactate dehydrogenase, a process that simultaneously oxidizes NADH to NAD^+^ (Fig. S5, *J* and *N*). We supplemented the cells with sodium pyruvate or AKB. Consistent with the effect of LbNOX overexpression, both treatments restored the NAD^+^/NADH ratio to control levels (Fig. S5, *K*–*M*, O–Q) and partially rescued the proliferation impairment (Fig. 4, *G*–*L*).To further investigate the role of BDH1 in tumor growth, we subcutaneously injected PaTu-8988t cells with or without BDH1 knockdown into mice. Tumors derived from BDH1-deficient cells exhibited slower growth, whereas supplementation with AKB significantly mitigated this impairment (Fig. 4, *M*–*O*). These results provide strong evidence that BDH1 is critical for maintaining the intracellular NAD^+^/NADH balance and sustaining cell proliferation and tumor progression in PDAC.

### BDH1-mediated NAD+/NADH homeostasis promotes PDAC cell proliferation *via* mitochondrial acetylation

The NAD^+^/NADH balance is essential for the proper functioning of most cellular metabolic reactions. Using metabolomics, we analyzed key NAD^+^-dependent processes, including glycolysis and TCA cycle (Fig. S6*A*), and assessed metabolic flux through metabolite ratios. Surprisingly, no significant differences were observed between BDH1 knockdown and control cells in the DHAP/3-PG ratio (Fig. S6*B*) or the Lactate/Pyruvate ratio (Fig. S6*C*). Similarly, no changes were detected in TCA cycle, including Isocitrate/α-ketoglutarate ratio (Fig. S6*D*), α-ketoglutarate/Succinyl-CoA ratio (Fig. S6*E*), and Malate/Citrate ratio (Fig. S6*F*). Additionally, evaluations of oxidative phosphorylation revealed that the oxygen respiratory capacity of siBDH1 cells was comparable to that of siCtrl cells (Fig. S6, *G*–*I*). These results strongly suggest that decreased NAD^+^/NADH in BDH1 knockdown cells has no significant effect on the metabolic level of PDAC cells.

**Figure 5:**
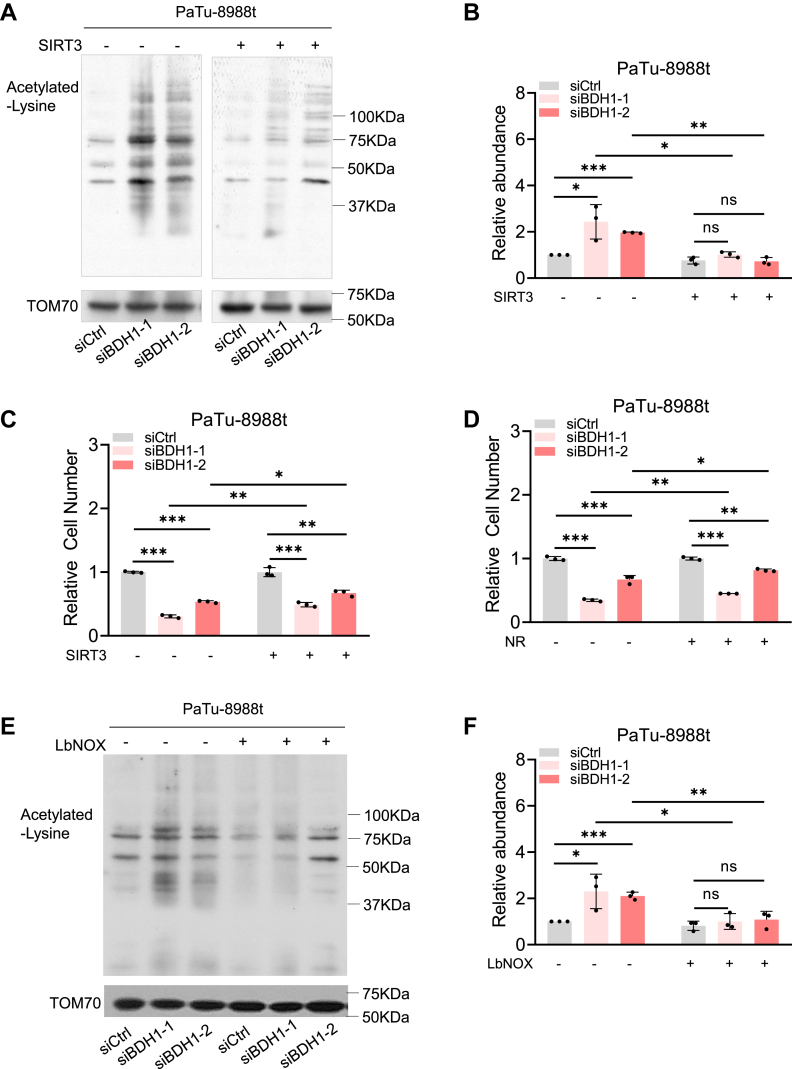
**BDH1-mediated NAD+/NADH homeostasis promotes PDAC cell proliferation *via* mitochondrial acetylation**. *A–B*, Western blot analysis of global mitochondrial protein acetylation in PaTu-8988t cells transfected with control siRNA, BDH1 siRNA, or BDH1 siRNA plus a SIRT3 expression vector (*A*), and corresponding quantitative analysis (*B*). *C*, PaTu-8988t cells transfected with control siRNA or BDH1 siRNA. Cell proliferation of overexpresing or not SIRT3, relative values were determined by comparing cells transfected with control siRNA to those transfected with BDH1 siRNA (mean ± SD, n = 3). *D*, PaTu-8988t cells transfected with control siRNA or BDH1 (bdh1) siRNA. Cell proliferation with and without NR supplementation, relative values were determined by comparing cells transfected with control siRNA to those transfected with BDH1 siRNA (mean ± SD, n = 3). *E–F*, Western blot analysis of global mitochondrial protein acetylation in PaTu-8988t cells transfected with control siRNA, BDH1 siRNA, or BDH1 siRNA plus a LbNOX expression vector (*E*), and corresponding quantitative analysis (*F*).

Mitochondrial deacetylase SIRT3 requires NAD^+^ as a substrate to regulate protein acetylation levels. In this study, we assessed the mitochondrial acetylation levels in BDH1 knockdown cells. The results revealed a significant increase in mitochondrial protein acetylation levels in PaTu-8988t cells following BDH1 knockdown (Fig. 5, *A* and *B*), a phenomenon that was also observed in MIA PaCa-2 cells (Fig. S7, *A* and *B*). Conversely, mitochondrial acetylation levels were significantly reduced in BDH1-overexpressing cells (Fig. S7*C*). These findings indicate that BDH1 plays a role in regulating mitochondrial acetylation in PDAC cells, likely through the modulation of NAD^+^ levels.

To explore whether mitochondrial acetylation levels are the cause of the proliferation of BDH1 knockdown cells, we overexpressed SIRT3 in PaTu-8988t BDH1 knockdown cells and found that high-expressing SIRT3 can reduce mitochondrial acetylation levels in both control cells and BDH1 knockdown cells, and the decrease in mitochondrial acetylation levels in BDH1 knockdown cells is more significant (Fig. 5, *A* and *B*). At the same time, cell proliferation experiments found that high-expressing SIRT3 can partially compensate for the decreased proliferation of BDH1 knockdown cells (Fig. 5*C*). Moreover, exogenous nicotinamide riboside (NR), a precursor of NAD^+^ and an activator of SIRT3, partially rescued the proliferation defect (Fig. 5*D*). Surprisingly, we found that overexpression of LbNOX significantly reduced the elevated mitochondrial acetylation levels observed in BDH1 knockdown cell (Fig. 5, *E* and *F*). This indicates that the increased mitochondrial acetylation level of PDAC is one of the reasons for the decreased proliferation of BDH1 knockdown cells.

Proteomic analysis of mitochondrial protein acetylation modification of siCtrl, siBDH1, and SIRT3 in PaTu-8988t cells. As anticipated, the acetylation levels of nearly all mitochondrial proteins decreased in SIRT3-overexpressing cells, while the acetylation levels of most mitochondrial proteins increased in BDH1 knockdown cells (Fig. S7*E*). These findings suggest that BDH1 promotes pancreatic cancer cell proliferation by regulating the NAD^+^/NADH balance and influencing the acetylation of mitochondrial proteins.

## Discussion

In recent years, with the advancement of metabolomics, cancer has increasingly been recognized as a metabolic disease characterized by dysregulated intracellular energy metabolism (19). PDAC is a highly invasive, desmoplastic tumor that is particularly vulnerable to chronic hypoxia and nutrient deprivation. Under these conditions, metabolic reprogramming is essential for tumor survival and progression (20). In addition to the well-established phenomena of aerobic glycolysis and glutamine addiction, recent studies have highlighted the significant role of ketone body metabolism in PDAC. Ketone body metabolism influences cellular homeostasis through mechanisms involving cellular metabolism, signal transduction, and epigenetic modifications. Gouirand *et al.* suggested that PDAC cells activate ketone body metabolism to utilize various nutrients for synthesizing β-hydroxybutyrate, which serves as an energy substrate, promoting tumor growth and progression (13). Similarly, Yang *et al.* demonstrated in KPC mice that a ketogenic diet combined with chemotherapy increased tumor NADH levels and synergistically inhibited tumor growth (21). This study found that ketone body metabolism-related genes are upregulated in PDAC tissues and that PDAC cells can synthesize and release HB and AcAc, suggesting a potential link between ketone body anabolism and PDAC pathogenesis. However, the mechanisms underlying the activation of ketone body anabolism during pancreatic cancer initiation and progression remain unclear.

Notably, this study found that exogenous ketone body supplementation had no significant effect on PDAC cell proliferation. While ketone bodies can serve as energy substrates, their role in PDAC may be influenced by the expression levels of intracellular ketone metabolism enzymes. Previous studies have shown that some cancer cells efficiently utilize ketone bodies for energy, whereas others exhibit resistance by downregulating key metabolic enzymes to suppress ketone oxidation. Our findings suggest that PDAC cells primarily rely on glycolysis and glutamine metabolism rather than ketone body oxidation. Consequently, exogenous ketone body supplementation may have a minimal impact on cell proliferation.

BDH1 is a key enzyme in ketone body metabolism, catalyzing the reversible conversion of AcAc to HB, with the concurrent interconversion of NADH to NAD^+^. While research on BDH1 in cancer remains limited, several studies have highlighted its potential role. For instance, Zhicheng Liu and Heng Zhang have shown that BDH1 expression is positively correlated with prognosis in hepatocellular carcinoma patients (22). Similarly, in acute myeloid leukemia, high BDH1 expression has been linked to the inhibition of tumor cell proliferation (23). In the present study, we observed elevated BDH1 expression in PDAC and found that BDH1 knockdown in PDAC cell lines (MIA PaCa-2, PaTu-8988t, and KPC cell line) suppressed cell proliferation. This suggests that BDH1 may play a significant role in PDAC progression. A series of proliferation assays revealed that BDH1 does not directly affect PDAC cell growth through HB or AcAc. Instead, BDH1 regulates PDAC cell proliferation by modulating the NAD^+^/NADH ratio. The HB/AcAc ratio is commonly used as an indicator of liver mitochondrial NAD^+^/NADH balance (24). This study further confirms that BDH1, a key enzyme in ketone body metabolism, plays a crucial role in maintaining the NAD^+^/NADH balance in PDAC cells. However, the precise contribution of ketone body metabolism to this balance in PDAC remains unclear and requires further investigation.

NAD^+^ is a crucial cofactor in enzymatic reactions and is involved in various metabolic processes (25). It is distributed in a highly compartmentalized manner across the cytoplasm, nucleus, and mitochondria, with distinct regulatory mechanisms in each subcellular compartment (19). Mitochondrial NAD^+^ plays a critical role in cancer biology, participating in essential processes such as the TCA cycle, oxidative phosphorylation, and fatty acid oxidation (26). Our study found that BDH1 knockdown significantly reduced the intracellular NAD^+^/NADH ratio; however, this change did not markedly affect the overall metabolic activity of the cells. PDAC cells may possess a robust metabolic regulatory mechanism that allows them to maintain energy homeostasis independently of BDH1-mediated NAD^+^/NADH balance. Additionally, compensatory metabolic pathways, such as glycolysis and glutamine metabolism, may help sustain NAD^+^ and NADH levels, thereby mitigating the impact of BDH1 on overall metabolism.

Proteomic modification studies indicate that BDH1 knockdown increases the acetylation levels of mitochondrial proteins. Given that NAD^+^ serves as a substrate for mitochondrial sirtuins (SIRT3, SIRT4, and SIRT5), which regulate protein acetylation (27). This finding suggests a potential link between BDH1 and mitochondrial deacetylation. Among these sirtuins, SIRT3 is the most potent mitochondrial deacetylase and plays a critical role in cancer development by modulating apoptosis, metabolism, and signaling pathways (28). For example, in head and neck squamous cell carcinoma (HNSCC), SIRT3 promotes cancer cell proliferation and migration by maintaining ROS levels, thereby driving tumor progression (29). Notably, over 35% of mitochondrial proteins contain acetylation sites, and more than 50% of proteins involved in key metabolic pathways—including energy production, fatty acid metabolism, glycolysis, and amino acid metabolism—are subject to acetylation (30). However, this study did not further investigate the specific target proteins affected by acetylation modifications. This study provides insight into the role of ketone body metabolism in PDAC cell growth, highlighting the importance of protein modification as a key mechanism.

## Experimental procedures

### Cell culture

hTERT-HPNE, PaTu-8988t, MIA PaCa-2, BXPC-3, CFPAC-1and PANC-1 were obtained from National Collection of Authenticated Cell Cultures (Chinese Academy of Sciences). all cell lines were identified by Short tandem repeat (Genetic Testing Biotechnology Corporation). Immortalized KPC cell line was provided by Dr Tao Xia from Zhejiang Provincial People's Hospital. The identity of the KPC cell line was confirmed through Sanger sequencing of the *Kras* and *Trp53* genes. The KPC cell lines were cultured in PRIM-1640 medium (Thermo Fisher Scientific) contains calf serum, while other cells were cultured in high-glucose DMEM medium (Sigma-Aldrich). For glucose-deprived culture conditions, the DMEM and calf serum were replaced with glucose-free Dulbecco's Modified Eagle's Medium (Sigma-Aldrich) and glucose-free dialyzed serum. All cells were cultured in a 37 °C incubator with 5% CO_2_ (Thermo Fisher Scientific) and treated with a low concentration of *mycoplasma* elimination reagent (0.5 μg/ml, Beyotime) to prevent *mycoplasma* contamination.

### Preparation of dialyzed serum

Serum was prepared by dialysis using dialysis membranes with a 1 kDa molecular-weight cutoff (Spectrum Labs) at 4 °C. The dialyzing buffer (151.425 g Tris-base, 37.275 g potassium chloride, and 400.314 g, added to 5 L of deionized water, pH = 7.35, all from Sigma-Aldrich) was replaced every 1 h for a total of 10 times, with the final replacement carried out overnight to maximize the removal of low-molecular-weight metabolites, as described previously (31).

### Mice

All animal studies were approved by the Animal Care Committee of Hangzhou Medical College (Production License Number: SYXK 2024–0002). Female nude mice (3–4 weeks) were purchased from Beijing Vital River Lab Animal Technology Co. Ltd. All animals were housed in groups under specific pathogen-free (SPF) conditions at the Experimental Animal Center of Hangzhou Medical College.

### Transfection and infection

siRNA and plasmid were transfected using Lipofectamine RNAiMAX (Thermo Fisher Scientific) and Lipofectamine 3000 (Thermo Fisher Scientific), respectively. Infectious lentivirus particles were generated by co-transfecting HEK293T cells with the target plasmid and two packaging plasmids (pMD2G and pSPAX2) at a ratio of 1:1:2. The culture medium containing infectious lentiviral particles was collected 48 h post-transfection, and filtered through a 0.45 μm filter (Millipore). PDAC cells infected with lentiviral particles were selected with 2 μg/ml puromycin (Beyotime) for 10 days before performing subsequent experimental procedures. The siRNAs used in this study were synthesized by Genepharma Biological Technology according to the following sequences: control siRNA, ACGTGACACGTTCGGAGAA; BDH1 siRNA1, GGATGACGAAATCCTTTCT; BDH1 siRNA2, GCCTGCGCTATGAGATGTA; bdh1 siRNA: GCAGGAAGTACTTCGATGAAA.

### RT and qPCR analysis

Total RNA was extracted using TRIzol reagent (Thermo Fisher Scientific) according to the manufacturer's instructions. For each sample, 1 μg of total RNA was reverse-transcribed into cDNA using HiScript II Q RT SuperMix (Novazan) in a 20 μl reaction system. Quantitative PCR (qPCR) was performed using ChamQ SYBR qPCR Master Mix (Novazan) on a Quantagene q225 system (KUBO-Tech AG, Switzerland). Each 20 μl qPCR reaction contained 10 μl of 2 × SYBR mix, 0.4 μl of each primer (10 μM), 2 μl of cDNA template (diluted 1:5), and 7.2 μl of nuclease-free water. The qPCR cycling conditions were: 95 °C for 30 s, followed by 40 cycles of 95 °C for 10 s and 60 °C for 30 s. Melt curve analysis was performed to confirm amplification specificity. The primers used for qPCR are listed in Table S1. All reactions were run in technical triplicate. Gene expression was normalized to β-actin, and relative mRNA levels were calculated using the 2ˆ-ΔΔCt method unless otherwise specified.

### Cell proliferation

Cells in optimal condition were digested into a single-cell suspension and counted. A total of 1 × 10^4^ cells were seeded per well in a 12-well plate, with a minimum of three replicates. After cells reached full attachment, they were trypsinized and counted using a NovoCyte 2040R flow cytometer (Agilent Technologies); this count was recorded as the cell number at day 0. Following 72 h of culture, cells were collected and counted again by flow cytometry, recorded as the cell number at day 3. The day 3 cell number was then normalized to the day 0 count for proliferation analysis. Additionally, AcAc and HB were added, alongside gene interference and various culture conditions, to assess their effects on cell proliferation.

### Colony formation assay

Cells were plated in triplicate in 6-well plates (Corning) at a density of 500 cells/well. The cells were cultured for 15 days, with medium refreshed every 3 to 4 days. After the culture period, the medium was removed, and 500 μl of 4% paraformaldehyde (Shanghai Lingfeng Chemical Reagent Co., Ltd) was added to the plates for 20 to 30 min. Colonies were then stained with 0.1% Crystal violet (Beyotime) for 20 to 30 min, and colonies whose diameters exceeded 0.5 mm were counted under a light microscope.

### Apoptosis analysis

Apoptosis was assessed using an Annexin V-EGFP/PI apoptosis detection kit (Jiangsu Keygen Biotechnology Co., Ltd), following the manufacturer's instructions. Cells were harvested and washed twice with PBS. After counting, 1 × 10^5^ cells were resuspended in 100 μl of binding buffer. Subsequently, 5 μl of Annexin V-EGFP and 5 μl of propidium iodide (PI) were added to the suspension. The samples were incubated in the dark at room temperature for 15 min. Apoptotic cells were analyzed using a NovoCyte flow cytometer (Agilent Technologies).

### IHC analysis and scoring

Pancreatic ductal adenocarcinoma (PDAC) tissue microarray was constructed by Dr Tao Xia from the Department of Pancreatic Surgery, Zhejiang Provincial People's Hospital, under protocol 2022QT010 approved by the hospital's Ethics Committee. All human studies were conducted in accordance with the principles of the Declaration of Helsinki. Paraffin-embedded tissue sections were first baked at 60 °C for 1 h to ensure adhesion, then deparaffinized twice in xylene for 10 min each and rehydrated through a graded ethanol series (100%, 95%, 85%, 75%) for 5 min each. Antigen retrieval was performed by heating the sections in sodium citrate buffer (10 mM, pH 6.0; Beyotime) at 95 to 100 °C for 20 min, followed by gradual cooling to room temperature. To block nonspecific antibody binding, sections were incubated with 5% goat serum (Beyotime) for 30 min at room temperature. Endogenous peroxidase activity was quenched by incubation with 3% hydrogen peroxide (Beyotime) for 10 min. Sections were then rinsed thoroughly with phosphate-buffered saline (PBS; Beyotime). Next, sections were incubated overnight at 4 °C with the primary antibody against BDH1 (1:200). After washing, an appropriate HRP-conjugated secondary antibody was applied at room temperature for 1 h. Visualization was performed using 3,3′-diaminobenzidine (DAB) chromogen solution (Beyotime) until the desired staining intensity was reached. Sections were counterstained with hematoxylin, dehydrated through graded ethanol, cleared with xylene (Changshu Yangyuan Chemical Co. Ltd), and mounted with neutral resin. Protein expression was assessed under a light microscope, and the integrated optical density (IOD) was quantified using Image-Pro Plus 6.0 (Media Cybernetics). The mean optical density (MOD), representing BDH1 intensity, was calculated as IOD/area.

### NAD^+^/NADH assay

NAD^+^/NADH ratio were measured using NAD^+^/NADH Assay Kit (Abcam), following the manufacturer's instructions. This assay utilizes an enzyme cycling reaction that specifically detects NAD^+^ and NADH with high sensitivity and is compatible with fluorescence detection in the red visible range, minimizing background interference. A total of 5 × 10^6^ cells were lysed in 100 μl of Lysis Buffer provided in the kit and incubated at 37 °C for 15 min. The lysates were then centrifuged at 1500 rpm for 5 min, and the resulting supernatants were collected for subsequent analysis. The supernatant was mixed with the detection reagents and transferred to a 384-well black plate for incubation in the dark for 30 min. Fluorescence was then measured at an excitation/emission wavelength of 540/590 nm using a SpectraMax iD3 Reader (Molecular Devices).

### AcAc and HB measurement

Cells in optimal condition were digested into a single-cell suspension and seeded into 60 mm dishes, targeting a confluency of 80% after 48 h. After 24 h of adherence, the medium was replaced with freshly prepared complete medium. The culture supernatant was collected after another 24 h. At the end of the incubation, culture supernatants were collected, centrifuged at 1000*g* for 5 min to remove cell debris. Based on the interconversion between AcAc and HB, which involves the oxidation or reduction of NADH/NAD^+^, the absorbance at 340 nm was measured before and after the enzymatic reaction to quantify NADH levels. Briefly, 10 μl of sample or standard solution was added to a 96-well plate. After the addition of the appropriate reaction buffer and enzymes, absorbance at 340 nm was recorded using a SpectraMax iD3 microplate reader (Molecular Devices) both before and after the enzymatic reaction. A standard curve is generated using the standard wells, and the HB or AcAc concentration in the sample wells is calculated.

### OCR assay

Oxygen consumption rate (OCR) assays were conducted using an Oxygraph-2k system (Oroboros), as previously described. Briefly, approximately 10ˆ6 cells were harvested and added to the chamber. Basal respiration was recorded, followed by the addition of oligomycin (1 μM; Sigma-Aldrich) to measure proton leak respiration. All respiratory data were normalized to cell number.

### Mitochondria Isolation

Cells were collected by centrifugation and resuspended in 2 ml of ice-cold homogenization buffer (0.35 M Tris-HCl, pH 7.8; 0.25 M NaCl; 0.05 M MgCl_2_). After incubation on ice for 2 min, the cell suspension was transferred to a pre-chilled glass Dounce homogenizer and homogenized until 80 to 90% of the cells were disrupted, as determined by trypan blue staining. Subsequently, 200 μl of equilibration buffer (3.5 mM Tris-HCl, pH 7.8; 2.5 mM NaCl; 0.5 mM MgCl_2_) was added and mixed gently. The homogenate was centrifuged at 1200×*g* for 3 min at 4 °C to remove nuclei and intact cells. The supernatant was collected and subjected to two additional centrifugation steps at 1200*g* for 3 min to ensure complete removal of debris. The final supernatant was then centrifuged at 15,000*g* for 2 min at 4 °C to pellet the crude mitochondrial fraction.

### Immunoblotting and antibodies

Total proteins were extracted from whole cells using RIPA lysis buffer (Cell Signaling Technology) supplemented with protease and phosphatase inhibitors (Cell Signaling Technology). Cells were lysed on ice for 20 min, followed by centrifugation at 14,000*g* for 10 min at 4 °C to collect the supernatant. Protein concentrations were determined using a BCA Protein Assay Kit (Thermo Fisher Scientific). Equal amounts of Proteins (20 μg) were separated using sodium dodecyl sulfate-polyacrylamide gel electrophoresis (SDS-PAGE) and transferred onto 0.22-μm polyvinylidene difluoride (PVDF) membranes (Bio-Rad, Hercules, CA) using a wet transfer system. Membranes were blocked with 5% non-fat milk in Tris-buffered saline containing 0.1% Tween-20 (TBST) for 1 h at room temperature, then primary antibodies were incubated overnight at 4  °C at the following dilutions: anti-BDH1 (1:1000; ProteinTech, Wuhan, China), anti-β-actin (1:2000; Santa Cruz Biotechnology, California, USA), anti-Flag (1:1000; ProteinTech), anti-SIRT3 (1:2000; Cell Signaling Technology, MA, USA), anti-kbhb (1:1000; PTM BIO, Hangzhou China), anti-pan acetylation (1:1000; Abcam). Quantification was carried out using the Gel-Pro Analyzer 4.0 (Media Cybernetics). After three washes with TBST (5 min each), membranes were incubated with appropriate horseradish peroxidase (HRP)-conjugated secondary antibodies (1:2000, Cell Signaling Technology) for 2 h at room temperature. Following washes, protein bands were visualized using enhanced chemiluminescence (ECL) reagent and imaged with a chemiluminescence imaging system. Band intensities were quantified using Gel-Pro Analyzer 4.0 software (Media Cybernetics). β-actin was used as a loading control to normalize protein expression levels.

### Animal experiments

As described previously. Cells were resuspended in a 1:1 mixture of Matrigel (Corning) and PBS and subcutaneously injected into the mid-posterior axillary region of 5–6-week-old nude mice using a needle. Each mouse was inoculated with 5 × 10ˆ6 cells. Once tumors formed, their size was measured once or twice weekly using a vernier caliper. Tumor volume was calculated using the formula: volume = (length × width × height) × 0.5236. After approximately 1 month, all mice were euthanized, and tumors were carefully excised. Tumors were then photographed and weighed.

### Untargeted metabolic profiling

To perform metabolite profiling experiments, samples were collected following the protocol provided by Metabo-Profle Biotechnology. Briefy, cells (1 × 10^7^ per sample) were collected and washed twice with cold PBS and then frozen in

liquid nitrogen for 15 min samples were sent to Metabo-Profle Biotechnology for metabolite measurements.

### TCGA and GTEx data analysis

Gene expression data from the combined TCGA and GTEx datasets were obtained from the UCSC Xena browser (https://xenabrowser.net/). Data comprising 178 pancreatic ductal adenocarcinoma samples and 171 normal pancreatic samples were extracted using custom scripts written in Strawberry Perl (version 5.28.1.1). Differential expression analysis and normalization of gene expression were performed using the DESeq2 R package (version 1.24.0), with statistical significance defined as *p* < 0.05.

### Statistical analysis

All experiments were conducted in triplicate and repeated independently at least three times. Data are presented as mean ± standard deviation (SD). Statistical analyses were performed using SPSS 21.0 (IBM), and *p*-values were calculated with an independent Student's *t* test. Statistical significance was defined as *p* < 0.05.

## Data availability

All data supporting the findings of this study are available within the article and its Supplementary Information files. Additional raw data and materials are available from the corresponding author upon reasonable request.

## Supporting information

This article contains supporting information.

## Conflict of interest

The authors declare that they have no conflicts of interest with the contents of this article.
