# Supplementary material for: β-hydroxybutyrate dehydrogenase promotes pancreatic cancer cell proliferation through regulation of the NAD+/NADH balance and mitochondrial acetylation
Source: J Biol Chem. 2025 Aug 28;301(10):110636. doi: 10.1016/j.jbc.2025.110636 (PMC12494558; doi:10.1016/j.jbc.2025.110636)
Supplement: Supplementary Figure Legend [file mmc1.docx]

**Supplementary Figure S1.**

A Single-cell RNA sequencing analysis of PDAC.

B Single-cell RNA sequencing analysis of KPC mice.

C HB concentration in the culture medium of PaTu-8988t treated with different concentrations of HB (0mM, 0.1mM, 0.5mM) for 24 hours under the conditions of 5 mM glucose and 1 mM glutamine.

D AcAc concentration in the culture medium of PaTu-8988t treated with different concentrations of AcAc (0mM, 0.1mM, 0.5mM) for 24 hours under the conditions of 5 mM glucose and 1 mM glutamine.

**Supplementary Figure S2.**

A PaTu-8988t cells were cultured under various conditions in the presence of HB. Western blot analysis was performed with the anti-KbHB and anti-β-actin.

B The relative levels of acetoacetate (AcAc) in PaTu-8988t cells after 24 hours of AcAc treatment under various culture conditions.

**Supplementary Figure S3.**

A-C Apoptosis analysis by flow cytometry of PDAC cells (PaTu-8988t, A; MIA PaCa-2, B; KPC cell line, C) transfected with control siRNA or siBDH1. The bar graphs on the bottom right show statistical analysis of apoptotic cell percentages (n = 3 per group).

**Supplementary Figure S4.**

A-C PDAC cells transfected with control siRNA or BDH1 (bdh1) siRNA. Western blot analysis was performed with the anti-KbHB and anti-β-actin, PaTu-8988t (A), MIA PaCa-2 (B), or KPC cell line (C).

D-F PDAC cells transfected with control siRNA or BDH1 (bdh1) siRNA. Cell proliferation levels after addition of NaCl and NaHB, NaCl (2mM), NaHB (2mM), relative values were determined by comparing cells transfected with control siRNA to those transfected with BDH1 (bdh1) siRNA (mean ± SD, n = 3), PaTu-8988t (D), MIA PaCa-2 (E), or KPC cell line (F).

G-I PDAC cells transfected with control siRNA or BDH1 (bdh1) siRNA. Cell proliferation levels after addition of LiCl and LiAcAc, LiCl (2mM), LiAcAc (2mM), relative values were determined by comparing cells transfected with control siRNA to those transfected with BDH1 (bdh1) siRNA (mean ± SD, n = 3), PaTu-8988t (G), MIA PaCa-2 (H), or KPC cell line (I).

**Supplementary Figure S5.**

A PaTu-8988t cells were transfected with control vector or vector expressing BDH1, Western blot analysis was performed with the indicated antibodies.

B NAD^+^/NADH ratio, relative values were determined by comparing cells transfected with control vector to those transfected with vector expressing BDH1 (mean ± SD, n = 3).

C-E PDAC cells transfected with control siRNA or BDH1 (bdh1) siRNA, Western blot analysis after overexpression of LbNOX were performed with the indicated antibodies.

F Schematic of LbNOX-catalyzed reaction

G-I PDAC cells transfected with control siRNA or BDH1 (bdh1) siRNA. NAD^+^/NADH ratio after overexpression of LbNOX, relative values were determined by comparing cells transfected with control siRNA to those transfected with BDH1 (bdh1) siRNA (mean ± SD, n = 3), PaTu-8988t (G), MIA PaCa-2 (H), or KPC cell line (I).

J Schematic diagram of the pyruvate reaction

K-M PDAC cells transfected with control siRNA or BDH1 (bdh1) siRNA. NAD^+^/NADH ratio after pyruvate supplementation, relative values were determined by comparing cells transfected with Control siRNA to those transfected with BDH1 (bdh1) siRNA (mean ± SD, n = 3), PaTu-8988t (K), MIA PaCa-2 (L), or KPC cell line (M).

N Schematic of AKB reaction

O-Q PDAC cells transfected with control siRNA or BDH1 (bdh1) siRNA. NAD^+^/NADH ratio after AKB supplementation, relative values were determined by comparing cells transfected with Control siRNA to those transfected with BDH1 (bdh1) siRNA (mean ± SD, n = 3), PaTu-8988t (O), MIA PaCa-2 (P), or KPC cell line (Q).

**Supplementary Figure S6.**

A Metabolic pathways and enzymes involved in NADH production using NAD⁺ as their cofactor, created with BioRender.com.

B-F Untargeted metabolomics analysis of PaTu-8988t cells with control siRNA or BDH1 siRNA, DHAP/3-PG (B), Lactate/Pyruvate ratio (C). Isocitrate/α-ketoglutarate ratio (D), α-ketoglutarate/Succinyl-CoA ratio (E) and Malate/Citrate ratio (F).

G-I Oxygen consumption rate (OCR) with control siRNA or BDH1 (bdh1) siRNA after successive addition of oligomycin (1 μM) (mean ± SD, n = 3), PaTu-8988t (G), MIA PaCa-2 (H), or KPC cell line (I).

**Supplementary Figure S7.**

A-B Western blotting of mitochondrial pan acetylation in MIA PaCa-2 with control siRNA or BDH1 siRNA, Tom70 used as loading control (A), quantitative statistical results (B).

C Western blotting of mitochondrial pan acetylation in PaTu-8988t cells with control vector or vector expressing BDH1, Tom70 used as loading control.

D PaTu-8988t cells transfected with control siRNA, BDH1 siRNA or BDH1 siRNA with vector expressing SIRT3. Western blotting analysis were performed with the indicated antibodies.

E Analysis of mitochondrial protein acetylation modification in PaTu-8988t cells with control siRNA, BDH1 siRNA and vector expressing SIRT3.
