# Supplementary material for: β-hydroxybutyrate dehydrogenase promotes pancreatic cancer cell proliferation through regulation of the NAD+/NADH balance and mitochondrial acetylation
Source: J Biol Chem. 2025 Aug 28;301(10):110636. doi: 10.1016/j.jbc.2025.110636 (PMC12494558; doi:10.1016/j.jbc.2025.110636)

Appendix Figure S1.

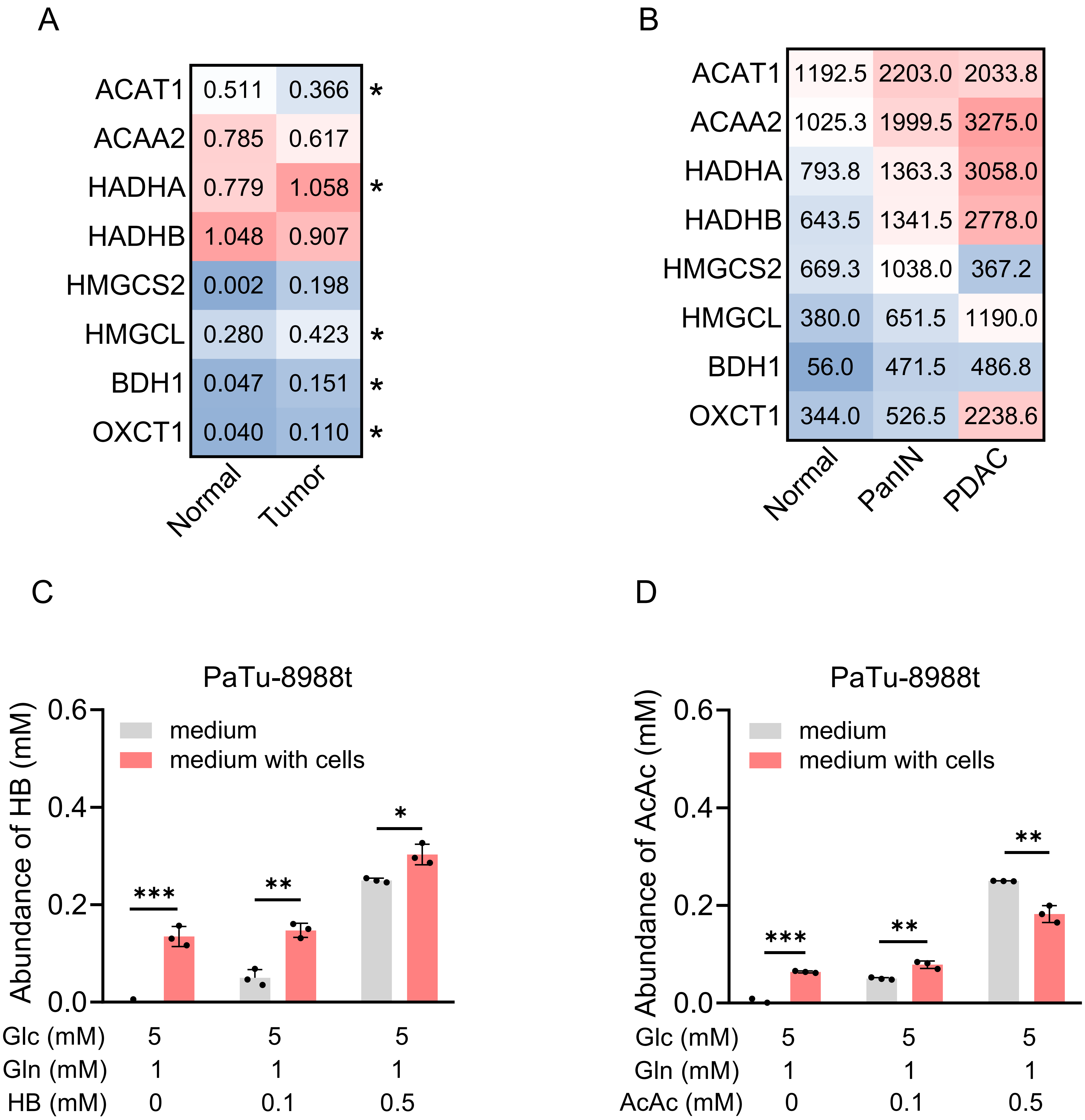

Appendix Figure S2.

A

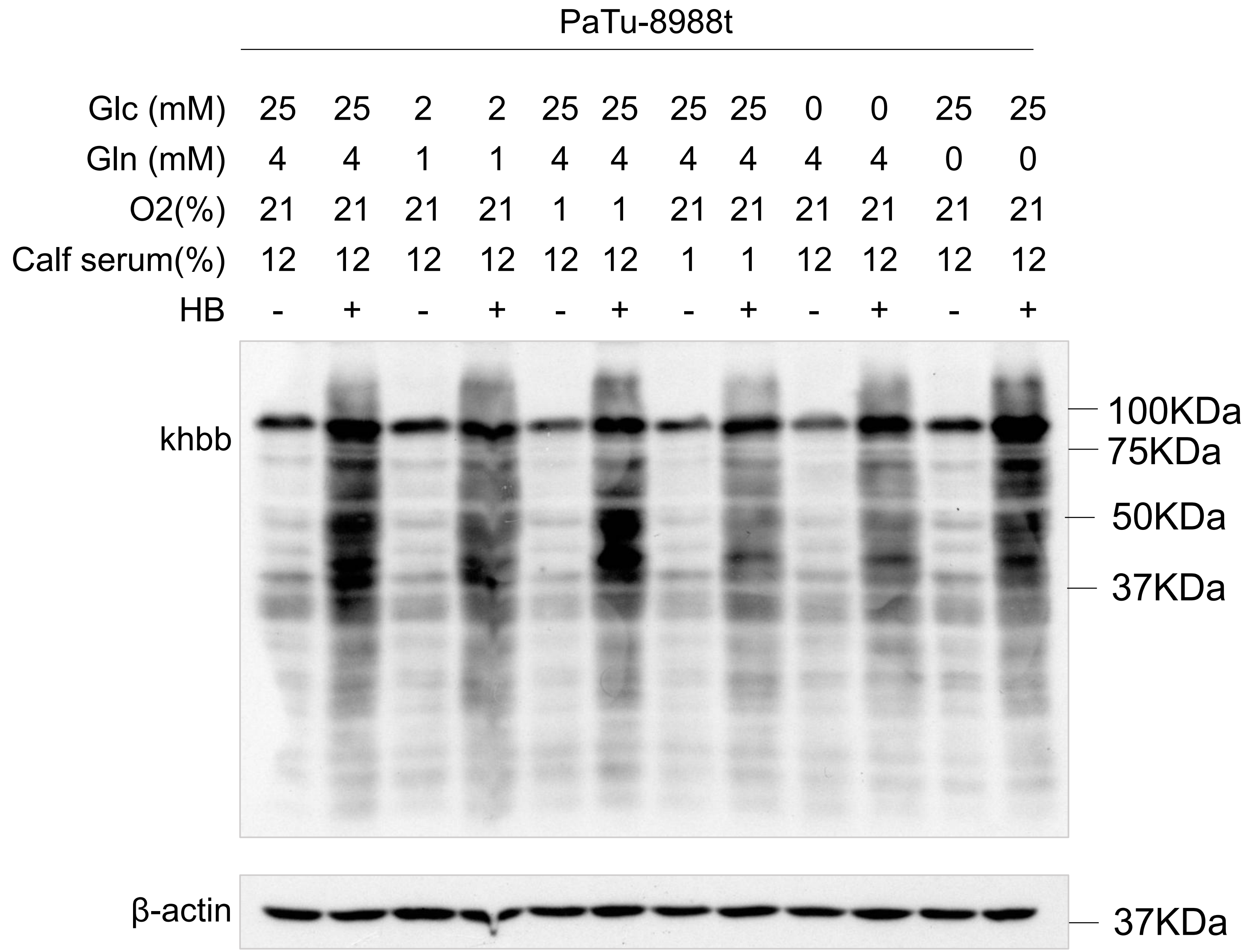

B

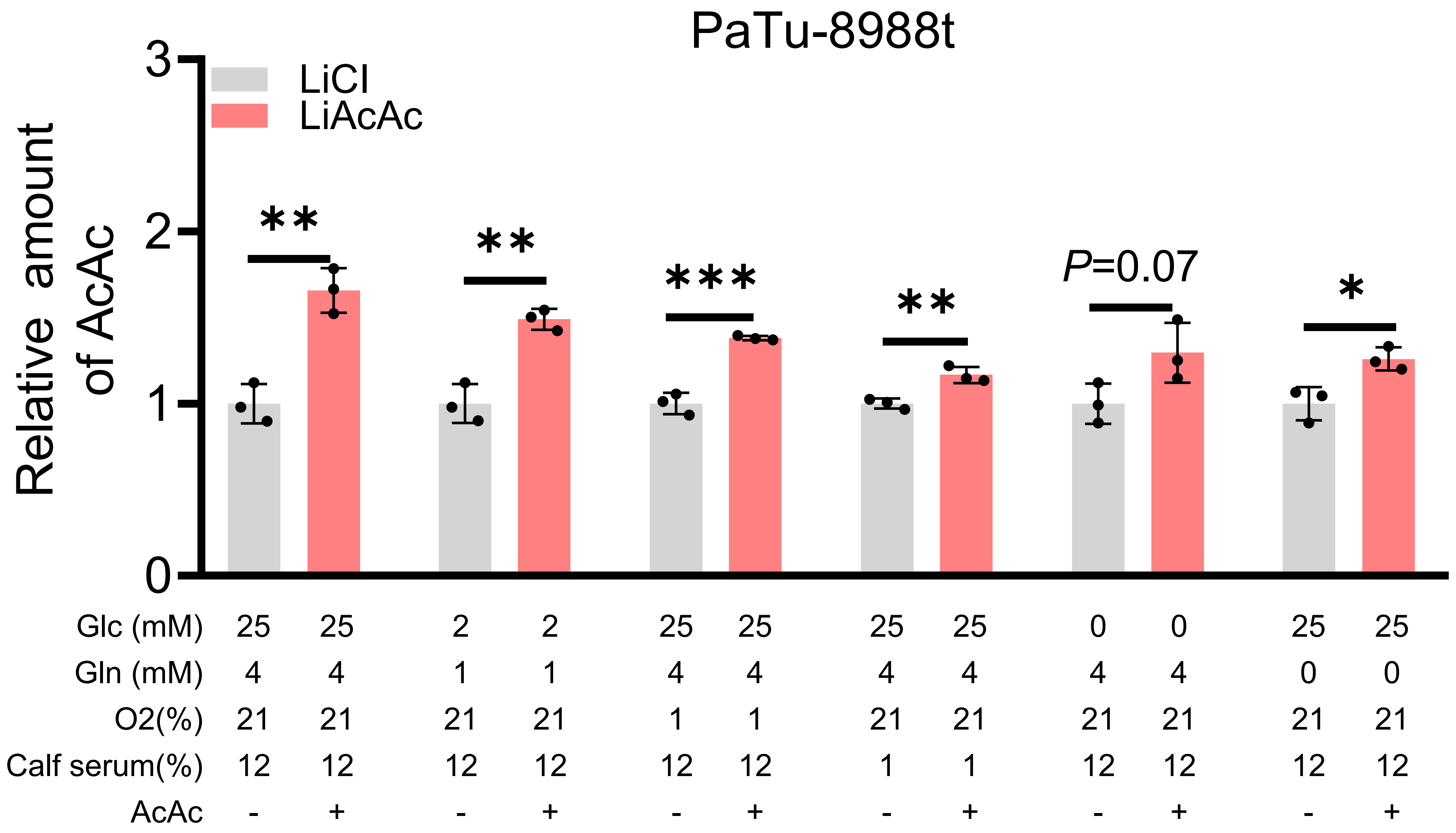

Appendix Figure S3.

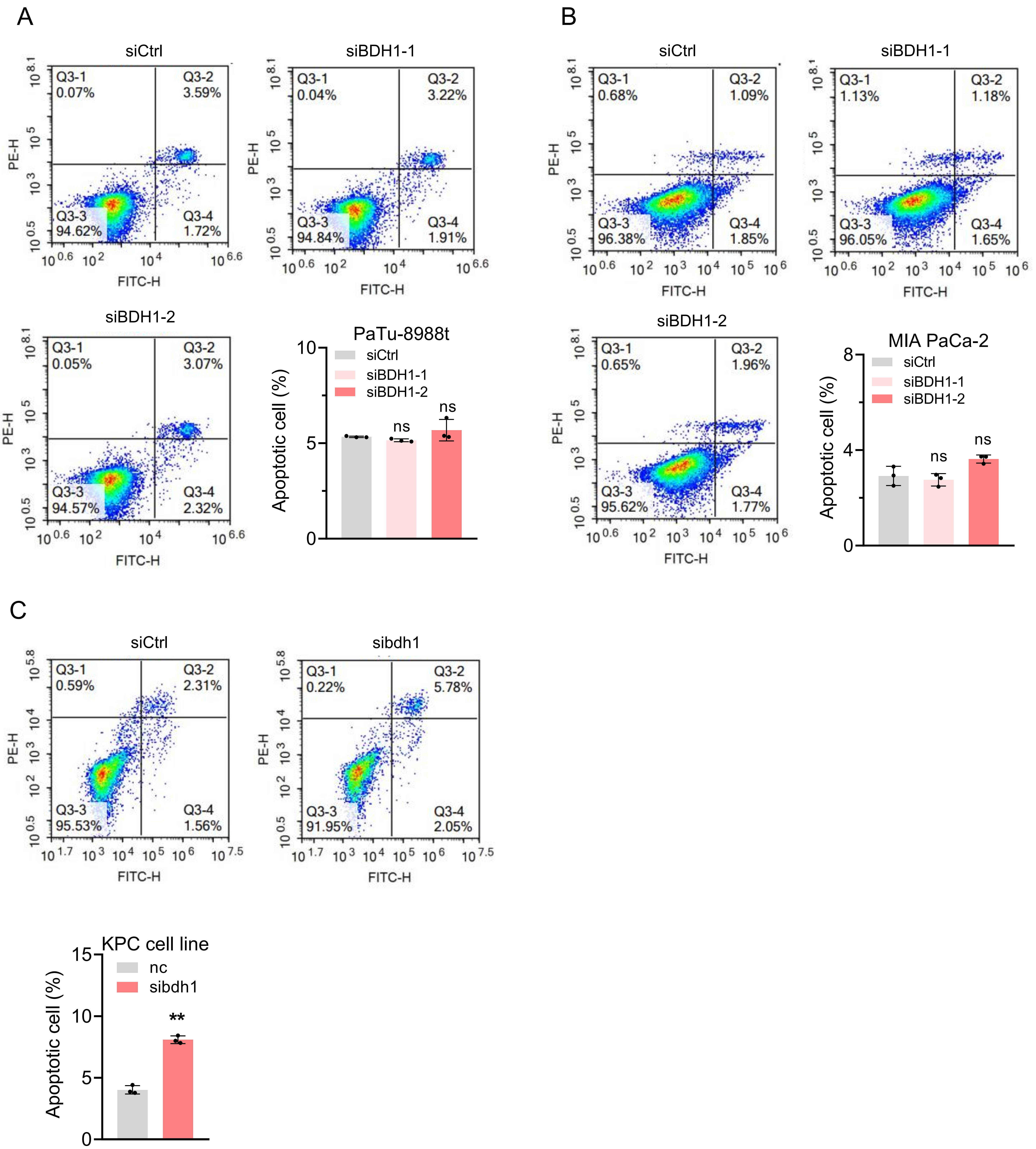

Appendix Figure S4.

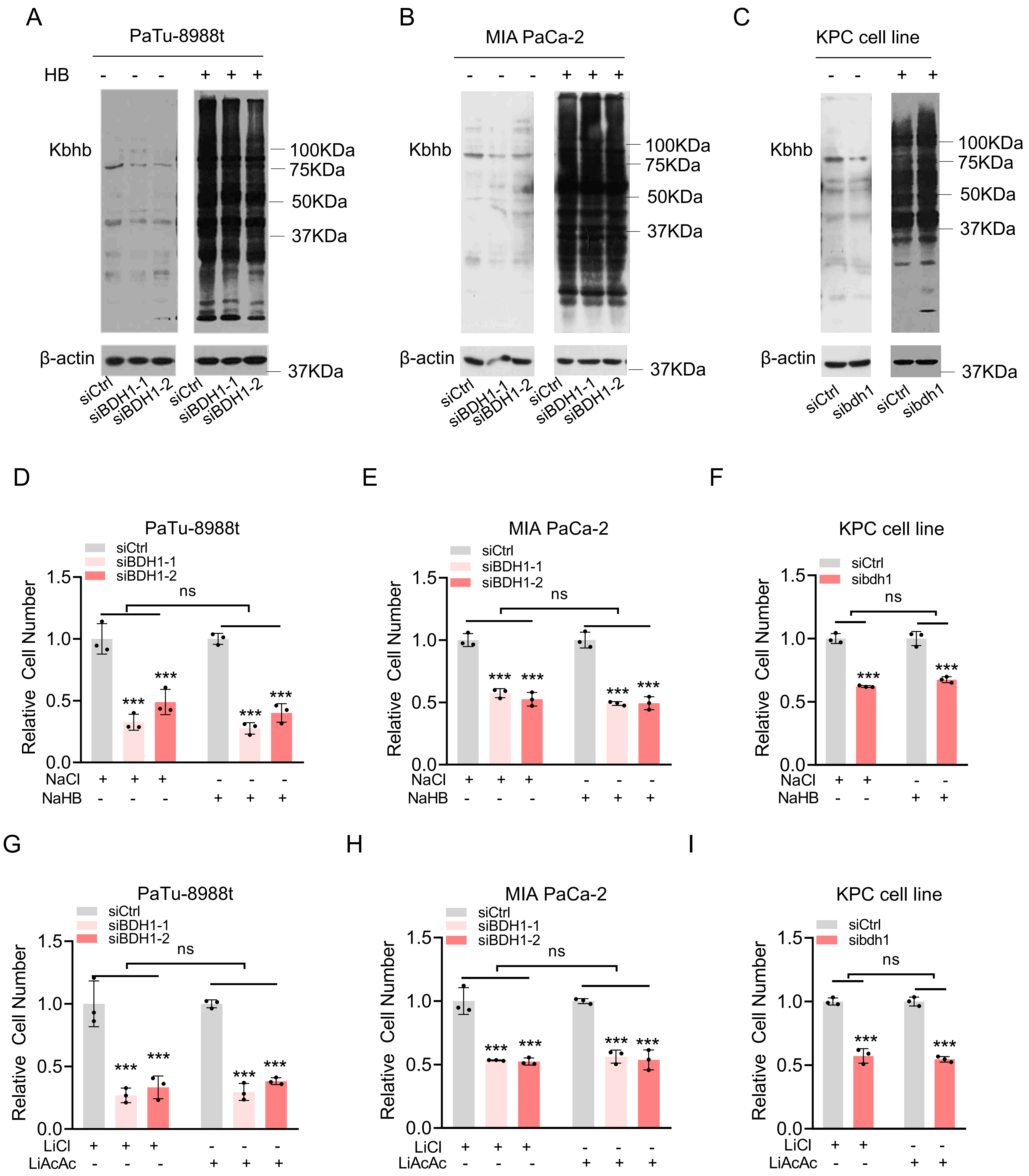

Appendix Figure S5.

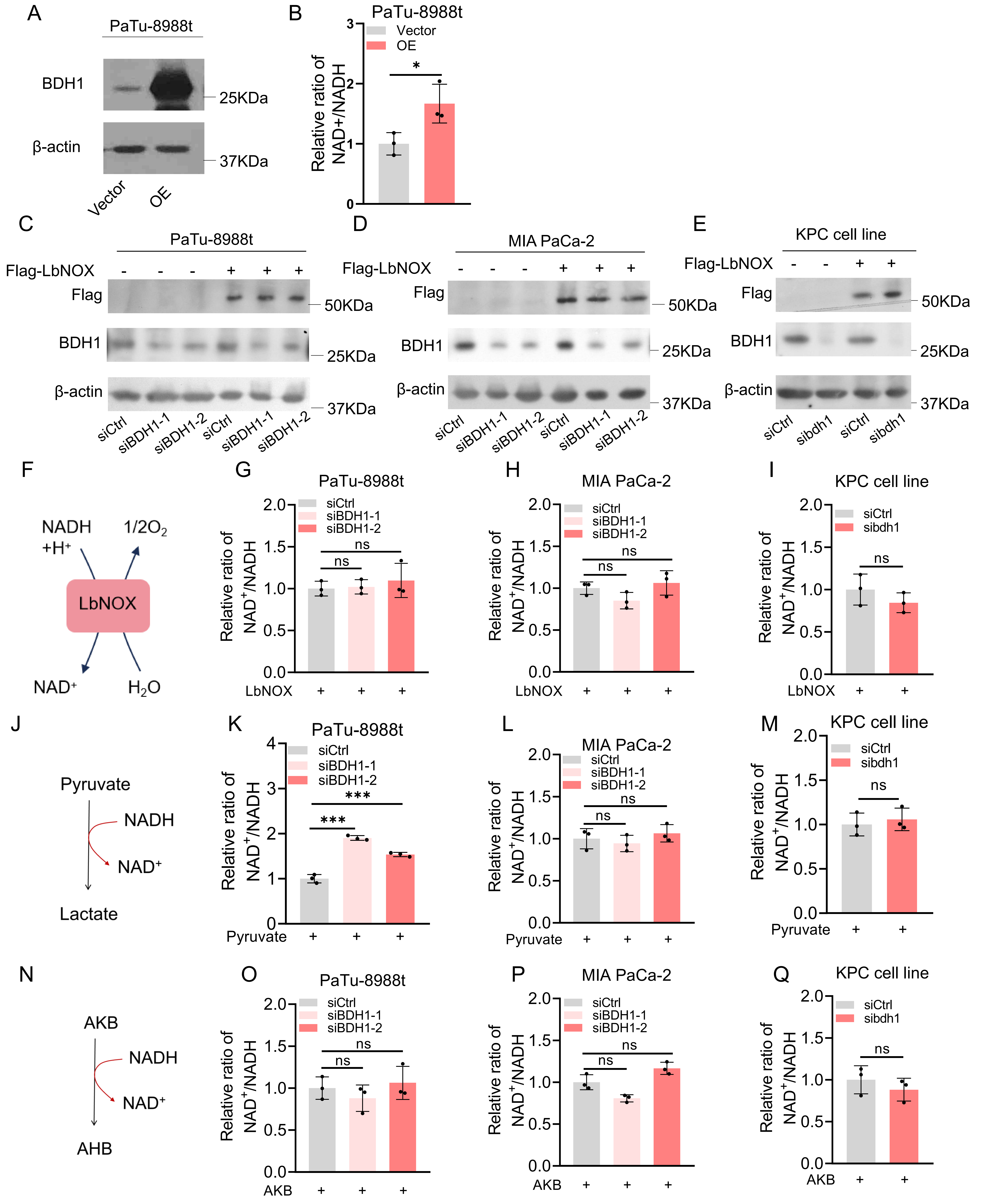

Appendix Figure S6.

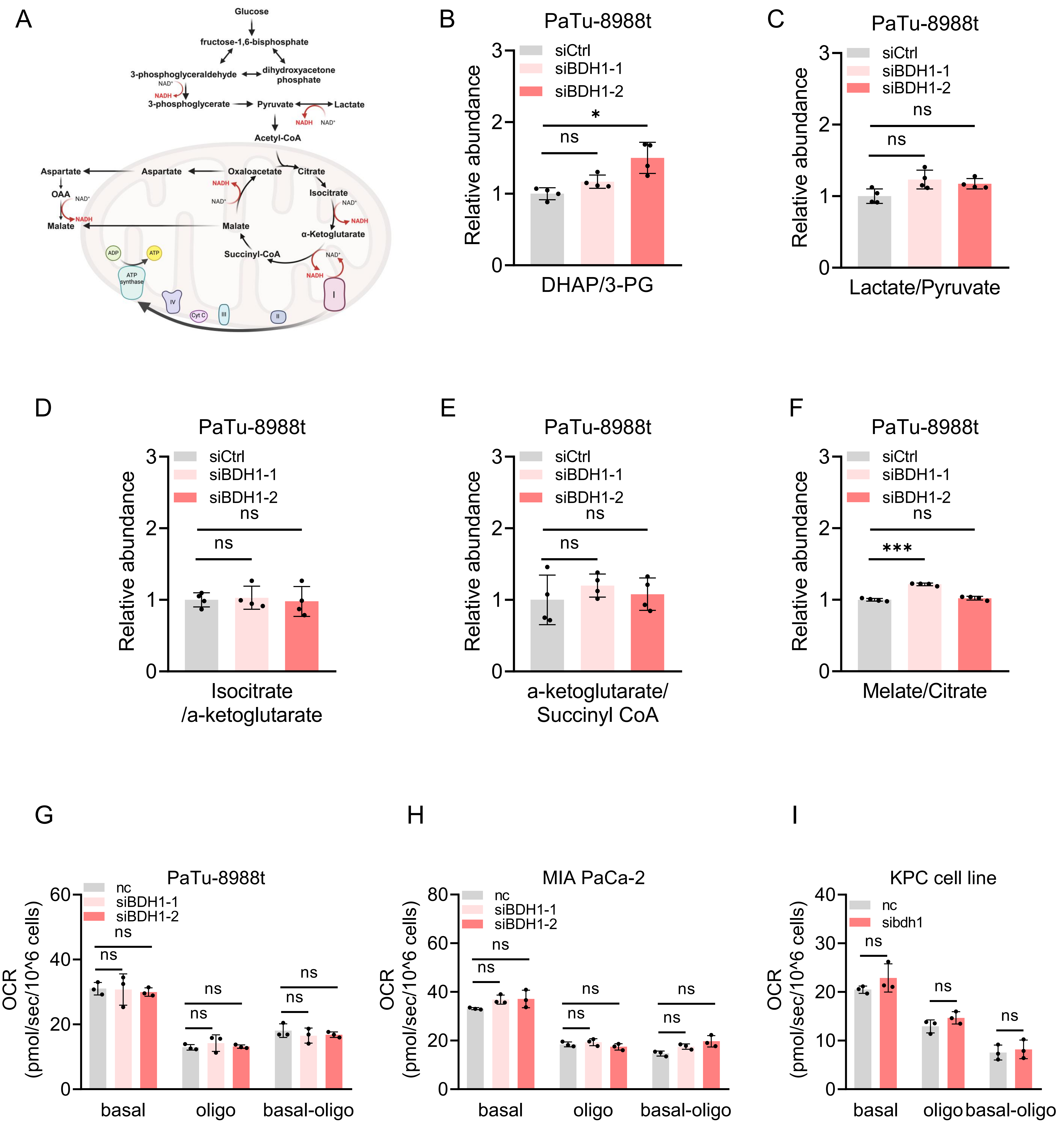

Appendix Figure S7.

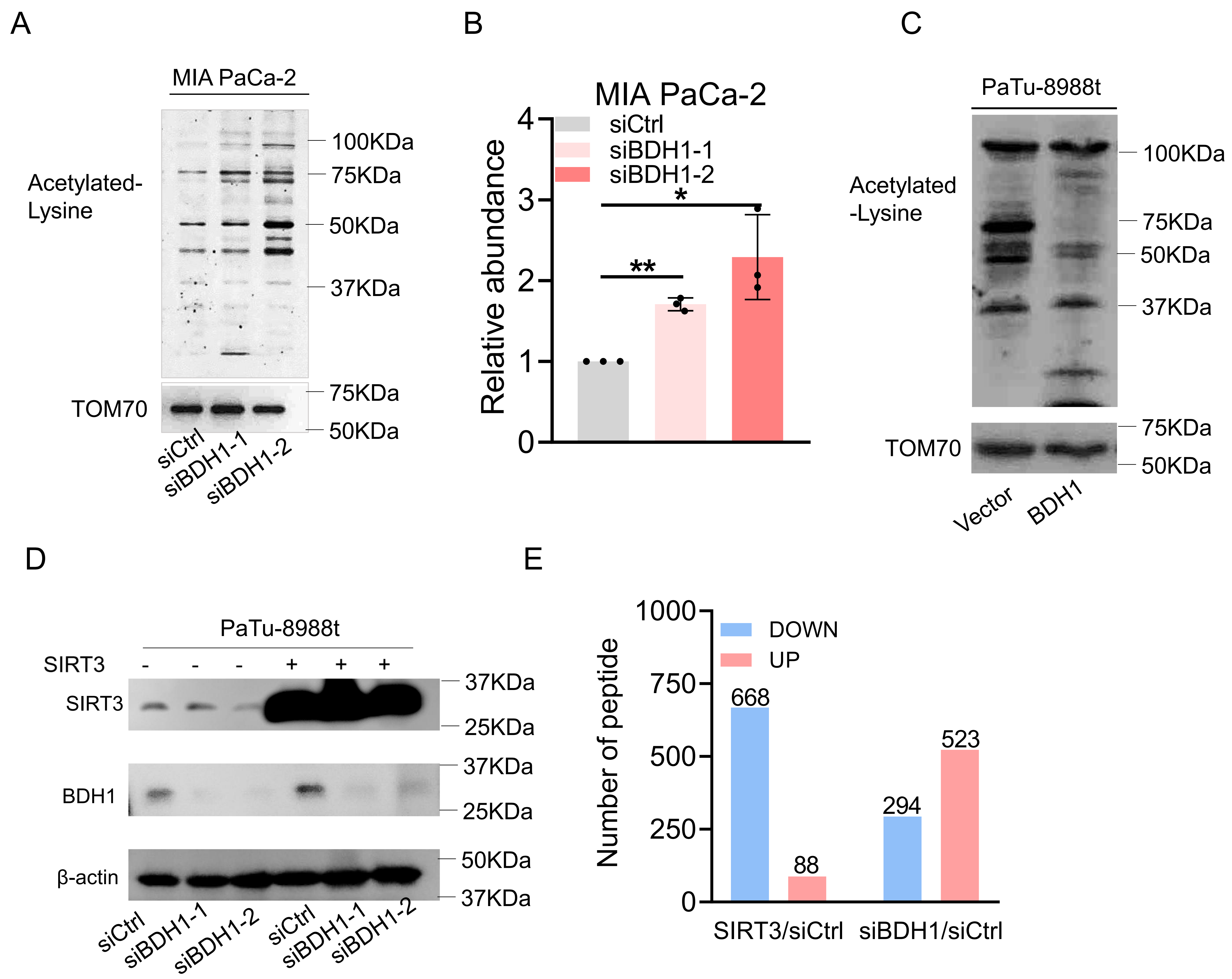

Supplement: Figures S1–S7 [file mmc2.pdf]
