# Supplementary material for: β-hydroxybutyrate dehydrogenase promotes pancreatic cancer cell proliferation through regulation of the NAD+/NADH balance and mitochondrial acetylation
Source: J Biol Chem. 2025 Aug 28;301(10):110636. doi: 10.1016/j.jbc.2025.110636 (PMC12494558; doi:10.1016/j.jbc.2025.110636)
Supplement: Table S1 [file mmc3.docx]

| **Table S1. Primers for qPCR** | |
| --- | --- |
| **Primer Name** | **Sequence (5’-3’)** |
| ACAT1 F | GGAGGCTGGTGCAGGAAATA |
| ACAT1 R | AGCAAGGAAAGGCTGCCTAA |
| ACAA2 F | CAGGGAATGCATCGGGTGTA |
| ACAA2 R | GCCCACAATTCTTGCCAGTG |
| HADHA F | CTGCCCAAAATGGTGGGTGT |
| HADHA R | GGAGGTTTTAGTCCTGGTCCC |
| HADHB F | CGGACGTCAGCCAAGATTCC |
| HADHB R | GTAGCTGGGAGGAACAGCTC |
| HMGCS2 F | CAGTTCCTGGGATGGGCTG |
| HMGCS2 R | TAACATCGATCCAAGGCCCG |
| HMGCL F | CCTCAGAGCTCTTCACCAAGAA |
| HMGCL R | GGAGACGTACCCCCGCAC |
| BDH1 F | GACTGCCTGCGCTATGAGAT |
| BDH1 R | TTCTTGGCGATGGCCTGAAT |
| OXCT1 F | ACCTGGTACAAGGGATGTGT |
| OXCT1 R | CAAAACCGTGGCACCATCAG |
| Actin F | GACCTGTACGCCAACACAGT |
| Actin R | AGTACTTGCGCTCAGGAGGA |
